# Gender Disparity in Grants and Awards at the National Institute of Health

**DOI:** 10.7759/cureus.14644

**Published:** 2021-04-23

**Authors:** Beenish Safdar, Sadiq Naveed, Amna Mohyud Din Chaudhary, Sundas Saboor, Muhammad Zeshan, Faisal Khosa

**Affiliations:** 1 Graduate Medical Education, John T Mather Memorial Hospital, Port Jefferson, USA; 2 Psychiatry, Hartford Hospital, Institute of Living, Hartford, USA; 3 Psychiatry, Nishtar Medical College and Hospital, Multan, PAK; 4 Department of Psychiatry, Khyber Medical University, Peshawar, PAK; 5 Department of Psychiatry, Rutgers University, Newark, USA; 6 Radiology, Vancouver General Hospital, Vancouver, CAN

**Keywords:** gender, nih funding, gender disparity, nih funding gender disparity, female researcher, academic promotion, academic productivity, research career

## Abstract

Objective

The National Institute of Health (NIH) supports the academic career of scientists across the United States (U.S.). It promotes and sponsors scientists in conducting wide-ranging clinical and basic science research. Depending on the duration, research type, and budget, there are various types of grants awarded by NIH. Despite considerable advancement in biomedical sciences, female researchers remain underrepresented in obtaining NIH funding. Through this study, we aim to highlight the gender trends in NIH funding and grants. By doing this, we aim to facilitate effective future policymaking to help achieve gender parity in NIH grants and awards.

Methods

The data were obtained from the NIH Research Portfolio Online Reporting Tool (RePORT). The extracted data by gender were tabulated showing percentages of females as Research Grant Investigators, Research Career Development Award Recipients and Kirschstein-National Research Service Award (NRSA) Trainees and Fellows, recipients of Research Grants, Research Project Grants (RPGs), and R01 equivalent grants including types 1 or 2, over two decades (1999-2019). Absolute percentage change was also calculated and included in the tables.

Results

The percentage of females as NIH Research Grant Investigators has increased at centers, research centers as well as for RPGs and Small Business Innovation Research and Small Business Technology Transfer (SBIR/STTR) programs. For Research Career Development Award Recipients and Kirschstein-NRSA Trainees and Fellows, the proportion of female pre-doctoral institutional trainees, post-doctoral fellows, post-doctoral institutional trainees, mentored research career awardees, and other research career awardees have steadily increased. However, there was a decrease in the percentage of female pre-doctoral fellow awardees. The percentage of females receiving all RPGs, R01-New (type 1) and R01-Renewal (type 2) grants has also decreased.

Conclusion

Despite an overall increase in the percentage of female researchers successfully receiving NIH grants and awards, they continue to lag compared to their male counterparts. With the increasing number of female doctoral graduates, it is imperative to address this disparity in NIH funding.

## Introduction

Despite the higher percentage of female doctoral graduates, there is a considerable gender gap in research leadership at federal government departments. More than half of the doctoral degrees in biology, psychology, and medical disciplines at the universities and research institutions in the U.S. are secured by females. This number of female doctoral graduates in the U.S. is expected to grow till the year 2029 [1]. A case-control analysis conducted on data extracted from the United States National Institutes of Health and other federal departments, including the Agency for Healthcare Research and Quality and Veterans Affairs showed that females comprised only 27% of the top research leadership at NIH and a lesser percentage in other departments. In addition to gender disparity at the faculty level, female scientists and researchers are less than one-third of the grantees at NIH [2]. For example, NIH intramural research program, an internal research program, has only 31% female research investigators [3].

The NIH is the world’s largest public funding agency and provides around $30 billion annually for biomedical research [4,5]. NIH's goal is to promote, facilitate, sponsor, and therefore enable major inventive disclosures, imaginative research methodologies, and their applications as a reason for eventually ensuring and improving general well-being. NIH comprises 27 research institutes and centers, each focusing on a specialized subdivision of biomedical sciences (e.g., National Institute of Mental Health, National Institute of Biomedical Imaging and Engineering, National Institute of Human Genome Research Institute, etc.). These institutes grant over 80% of their budget to support research initiatives worldwide, including 50,000 competitive awards to more than 300,000 scientists at 2,500 colleges, medical schools, and other research foundations [6].

The NIH grants are specified by an activity code representing the type of research funded [7]. The individual eligibility for grants varies from pre-doctoral students on research training grants to investigators with extensive experience that run large research centers. This study aims to highlight the gender trends of NIH grant support and characterize potential explanations for existing gender differences. Through this study, we aim to facilitate future policy-making to achieve gender parity in NIH funding.

## Materials and methods

Our methodology has been validated in recent publications [3,8]. Institutional Review Board approval was not required for this retrospective study. Data were obtained from the publicly available data at NIH Research Portfolio Online Reporting Tools (RePORT) - NIH Data Book [9]. We utilized the NIH grant and funding reports for the consecutive fiscal years 1999 to 2019.

Variables and data analysis

The percentage of females as Research Grant Investigators (RGIs) and the differences by mechanism, i.e., Research Project Grants (RPGs), at Centers, for other research, as Research Career and Small Business Innovation Research/Small Business Technology Transfer (SBIR/STTR) were examined, from the year 1998 to 2019. Similarly, the percentage of females (by Activity Code and Career Stage) as Research Career Development Award Recipients and Kirschstein-NRSA Trainees and Fellows were compared over the study period to examine the temporal trends. The changes in the percentage of females receiving Research Grants were also surveyed, as well as the average funding of these research grants in current dollars (amount received unadjusted for inflation) and constant dollars (amount received after adjustment for inflation) was also compared between genders [10].

The absolute percentage change was calculated to highlight the trends of female representation under the observed categories. This was a descriptive study so no statistical tests of significance were conducted.

## Results

Research grants are defined as extramural awards made for research centers, research projects, small business innovation research/small business technology transfer (SBIR/STTR), and other research grants. Between the years 1998 and 2019, there was an absolute increase of 11% in the percentage of females receiving research grants (Table 1). Furthermore, there has been a substantial increase in females’ percentage as Research Grant Investigators (RGIs) in a research career, with an absolute increase of 18%. It is followed by an increase for female RGIs in the centers by 15%, females conducting other research by 14%, and a 12% increase in females receiving the RPGs in these two decades (Table 2). The least absolute increase of 5% was seen in the percentage of females receiving the SBIR/STTR. But overall, the increase in each category for females was promising and reassuring.

**Table 1:** Research grants: awards by gender and percentage to females. Research grants are defined as extramural awards made for research centers, research projects, Small Business Innovation Research/Small Business Technology Transfer (SBIR/STTR), and other research grants. Research Grants are defined by the following activity codes: R, P, M, S, K, U (excluding UC6), DP1, DP2, DP3, DP4, DP5, D42, & G12.

Year	Females	Males	Percentage to Females
1998	7,362	24,877	23%
1999	8,040	26,753	23%
2000	8,713	28,320	24%
2001	9,386	29,890	24%
2002	10,199	31,801	24%
2003	11,080	33,416	25%
2004	11,693	34,200	25%
2005	11,998	33,870	26%
2006	11,929	33,107	26%
2007	12,266	33,551	27%
2008	12,575	32,987	28%
2009	12,546	31,884	28%
2010	12,635	31,667	29%
2011	12,838	31,183	29%
2012	13,025	30,768	30%
2013	12,522	29,553	30%
2014	12,516	28,843	30%
2015	12,755	28,703	31%
2016	13,355	29,287	31%
2017	13,842	29,709	32%
2018	15,207	30,648	33%
2019	16,343	31,558	34%
Absolute Change (%)			+11%

**Table 2:** Research grant investigators: percentage of females, by mechanism.

Year	RPGs	Centers	Other Research	Research Career	SBIR/STTR
1998	22%	11%	25%	35%	17%
1999	23%	12%	25%	33%	18%
2000	23%	12%	26%	35%	17%
2001	23%	13%	27%	35%	18%
2002	24%	16%	28%	36%	18%
2003	24%	17%	28%	37%	18%
2004	25%	17%	29%	39%	19%
2005	25%	17%	28%	40%	18%
2006	26%	18%	27%	42%	19%
2007	26%	18%	26%	43%	18%
2008	29%	19%	29%	44%	17%
2009	29%	18%	31%	44%	19%
2010	29%	20%	32%	45%	19%
2011	30%	20%	34%	46%	18%
2012	30%	20%	34%	45%	20%
2013	31%	21%	34%	46%	20%
2014	31%	22%	35%	47%	19%
2015	31%	22%	36%	48%	19%
2016	32%	23%	36%	50%	21%
2017	32%	25%	36%	50%	19%
2018	33%	26%	38%	51%	21%
2019	34%	26%	39%	53%	22%
Absolute change (%)	+12%	+15%	+14%	+18%	+5%

The overall percentage of females receiving Research Career Development Award Recipients and Kirschstein-NRSA Trainees and Fellows showed an increase except in the category of pre-doctoral fellows where females had an absolute decrease of 18% (Table 3). All other categories had an increased representation of females, with the greatest increase seen in Mentored Research Career Awardees (+28%) and the least increase in females as post-doctoral fellows (+9%) (Table 3).

**Table 3:** Research Career Development Award recipients and Kirschstein-NRSA Trainees and Fellows: percentage of females, by activity code and career stage. Pre-doctoral fellowships include activity codes F30 and F31. Post-doctoral fellowships include activity codes F32. Mentored Research Career Awards include activity codes: K01, K07, K08, K22, K23, K25, K99, KL1, and KL2. Other Research Career Awards are defined as any other K activity code not included in Mentored Research Career Awards. Kirschstein-NRSA Training Grants include activity codes T32, T34, T35, T36, T90, TL1, TL4, and TU2. Not all of these activities may be in use by NIH every year.

Year	Pre-Doctoral Fellows	Pre-Doctoral Institutional Trainees	Post-Doctoral Institutional Trainees	Post-Doctoral Fellows	Mentored Research Career Awardees	Other Research Career Awardees
1990	73%	45%	38%	41%	25%	31%
1991	66%	45%	40%	41%	25%	31%
1992	64%	45%	42%	41%	26%	32%
1993	58%	48%	42%	41%	27%	30%
1994	58%	49%	43%	42%	29%	31%
1995	59%	49%	43%	42%	30%	30%
1996	59%	49%	45%	43%	32%	31%
1997	57%	49%	45%	43%	34%	33%
1998	57%	49%	47%	41%	36%	32%
1999	59%	50%	46%	42%	36%	30%
2000	60%	51%	47%	43%	37%	30%
2001	59%	53%	47%	46%	37%	30%
2002	58%	54%	48%	45%	39%	29%
2003	59%	54%	48%	43%	39%	29%
2004	59%	55%	49%	42%	41%	31%
2005	60%	55%	50%	43%	41%	31%
2006	60%	55%	51%	45%	43%	32%
2007	63%	52%	51%	44%	45%	34%
2008	63%	53%	52%	46%	46%	33%
2009	61%	52%	51%	48%	46%	33%
2010	61%	52%	54%	49%	47%	35%
2011	58%	52%	54%	49%	47%	37%
2012	57%	52%	55%	49%	47%	35%
2013	57%	52%	57%	50%	47%	37%
2014	55%	52%	56%	50%	48%	40%
2015	55%	52%	55%	52%	49%	41%
2016	54%	54%	55%	50%	51%	42%
2017	54%	54%	56%	49%	51%	47%
2018	54%	55%	58%	50%	52%	45%
2019	55%	56%	58%	50%	53%	48%
Absolute Change (%)	−18%	+11%	+20%	+9%	+28%	+17%

When comparing the funding of research grants, the total funding amount for females was less each year since 1998, with males receiving higher funding amounts in both current and constant dollars (Table 4). Females received higher funding in 2018 for SBIR/STTR than males when the analysis was done for funding by category and comparing the funding for the years 2018 and 2019. In comparison, all other funding by mechanism had greater funding received by males (Table 5).

**Table 4:** Research grants: average funding in current dollars, by gender. Research grants are defined as extramural awards made for research centers, research projects, Small Business Innovation Research/Small Business Technology Transfer (SBIR/STTR), and other research grants. Research grants are defined by the following activity codes: R, P, M, S, K, U (excluding UC6), DP1, DP2, DP3, DP4, DP5, D42, & G12. Current dollars and constant dollars represent average costs. Constant dollars were computed using 1998 as the base from the Biomedical Research and Development Price Index (BRDPI) based on the latest fiscal year. Constant dollar figures are not yet available for FY2019.

	Constant Dollars		Constant Dollars (1998)	
Year	Females	Males	Females	Males
1998	$241,565	$302,196	$241,565	$302,196
1999	$266,728	$325,240	$258,959	$315,767
2000	$291,697	$352,750	$272,614	$329,673
2001	$312,978	$381,833	$281,962	$343,993
2002	$330,169	$403,047	$289,622	$353,550
2003	$342,543	$419,264	$290,291	$355,308
2004	$347,859	$433,881	$282,812	$352,749
2005	$353,779	$450,304	$278,566	$354,570
2006	$360,271	$452,813	$270,881	$340,461
2007	$365,276	$453,443	$263,965	$327,679
2008	$372,385	$461,483	$257,688	$319,343
2009	$392,299	$486,906	$270,168	$335,322
2010	$404,801	$501,715	$270,340	$335,063
2011	$408,257	$503,126	$264,164	$325,549
2012	$421,385	$507,279	$269,624	$324,584
2013	$410,095	$496,342	$257,959	$312,210
2014	$441,835	$525,504	$265,811	$316,147
2015	$449,262	$528,625	$264,369	$311,070
2016	$472,889	$547,136	$272,344	$315,104
2017	$496,360	$566,037	$278,613	$317,724
2018	$505,271	$579,673	$276,200	$316,871
2019	$530,694	$599,511	NA	NA

**Table 5:** Research grants: average funding, by mechanism and gender, for the years 2018 and 2019.

	2018		2019	
Type	Females	Males	Females	Males
All other research grants	$559,731	$688,200	$653,784	$801,882
Research Career Awards	$176,807	$193,755	$177,109	$199,361
Research centers	$1866,092	$2277,084	$1,908,165	$2,384,220
Research project grants	$519,030	$544,772	$547,462	$557,997
SBIR/STTR	$511,814	$500,882	$513,805	$536,591
Total average	$505,271	$579,673	$530,694	$599,511

## Discussion

This article reviewed the gender differences in NIH funding and grants, contributing to impediments for ensuring a diverse faculty. NIH funding is considered academically prestigious and can often be critical in retaining and promoting faculty [11]. Our data displays the trends in the advancement of females in research careers in the last two decades. Undoubtedly, females have made progress as research investigators, awardees of small business grants, and overall RPGs. However, the percentage of female pre-doctoral (K01) awardees is not comparable to their rising number in the U.S. Therefore, females are not well represented in research careers at the NIH, compared to their male counterparts.

The previous studies exploring the effects of gender on NIH funding have shown mixed results [12,13]. A retrospective cohort study (1997-2007) showed that less than one-quarter of K award recipient females were able to receive a higher award within five years as compared to their male counterparts [11]. A "spillage issue" in the early pipeline of academic career, even among those who want research vocations, is more prominent for females [11]. Another study analyzed the NIH data regarding research and training grants supported in 2008. It found that females and males were generally equally successful at all career stages. However, males with previous experience as NIH grantees had higher application and funding rates than females at similar career points [12]. Female MDs, as first-time investigators, had a 20% success rate compared to males (24%), and as experienced investigators, females were 32% successful compared to 36% of experienced male applicants [13]. These findings were also corroborated in a study exploring the NIH grant funding in the discipline of radiology. It was reported that there is a significant gender difference in mean NIH grants awarded to radiology investigators for 2016-2019 inclusive ($619,807.00 for male Ph.D. investigators vs. $158,486.00 for female Ph.D. investigators) [14].

A study examining the gender-specific NIH grant application rates found that the number of females submitting grants to NIH was less than males (56% vs 62%), female investigators submitted no more than one grant, applied for lesser years of funding than males (3.1 years vs 3.4 years), smaller budget ($115,325 vs $150,000), the success rate of grants and resubmission of the grants was significantly higher for males than females (41% vs 44%, and 43% for females and 51% for males, respectively). There is gender parity in grant success rates at higher academic ranks, while some gender disparity was noticed in lower academic ranks [15]. Findings from another study raised the possibility of gender bias in the NIH peer review process of grants [16].

Gender discrimination also persists in academic publishing and institutional policies [17,18]. A retrospective analysis (1997-2004) of prominent medical research journals showed that the percentage of females as first and last authors of original studies increased, but their percentage remained low as authors of original articles and guest editorials compared to males [16]. Another study found that after adjustment for publications and seniority, the funding gap between the genders was no longer significant (P = 0.08 and P = 0.16 for females and males, respectively), suggesting that seniority and publications play an important role in award size [3]. The Buddeberg-Fischer cohort found that female physicians received significantly less mentoring [19]. Mentorship is pivotal for career guidance, research productivity, and grant success [19]. Unfortunately, females have greater difficulty finding mentors than males especially since the advent of the ME 2 movement which has given voice to many on one hand, has also had unintended consequences [20].

There were several limitations to our study because the information mirroring applicants' academic reputation and credentials were not accessible for analysis, we cannot rule out the likelihood that gender disparities in features related to academic productivity (age, authorship position, academic rank) brought about differential candidate assessments in our investigation [21]. Also, we did not have access to individual award amounts, so we cannot conclusively remark on whether differences in award amounts contribute to the observed gender differences in various award achievements. A portion of the observed gap may also originate from professional choices made by males and females with some theorizing that females may have different priorities regarding the balance between work and other pursuits, devote most of their time in teaching and clinical activity than to research [22].

Lastly, our study shows that although we are progressing towards increased participation of females in NIH programs, further policies and actions are required to circumvent and mitigate the remaining gender differences. These should include the development of new initiatives, in addition to continuing current programs, while carefully monitoring progress to ensure that resources are available to allow scientists to achieve successful and meaningful research careers and to improve the health of our nation.

## Conclusions

It is important to address the underrepresentation of women at the world’s largest research centers like NIH. Despite considerable advancement in gender equity, women are underrepresented as NIH grant recipients and awardees. This disparity is more evident for the women's pre-doctoral fellow awards. Although the trends show an improvement in female representation and funding at the NIH, the progress has been slower when compared to the dramatic increase in female doctoral candidates in recent years. Further studies are needed to explore whether the differences in NIH awards and funding are due to lower application success rates or a lower proportion of females applying for these grants. Nevertheless, concrete steps are required to ensure and encourage female funding and progress the research careers of females at the NIH.
